# 

**Published:** 1920-05

**Authors:** 


### ANNOUNCEMENTS

### Northern Ohio Dental Association

The next meeting will be held in Cleveland, June 7, 8 and 9, 1920, in the Hotel Statler. It is also planned to hold a special post-graduate course immediately following the meeting, June 10th and 11th, by special arrangement with special instructors.

### Graduation of Dental Hygienists

The Rochester Dental Dispensary graduated in its mid-semester term fifteen dental hygienists, February 1, 1920. The mid-term graduation will not be continued after this year.

### Michigan State Dental Meeting

The annual meeting was held in Detroit, April 12th to 14th. An exceptionally interesting meeting was held, although the program was not characterized by the usual parade of out-of-state papers and the ''Detroit Clinic Club.'' The papers were Michigan men largely as were the clinical contributors. It was really refreshing and enjoyable. Dr. Bates was elected president and R. W. Binting president-elect for the ensuing year. Dr. Claude S. Larned, of Battle Creek, was re-elected secretary.

